# Assessing Program Efficiency: A Time and Motion Study of the Mental Health Emergency Care — Rural Access Program in NSW Australia

**DOI:** 10.3390/ijerph110807678

**Published:** 2014-07-31

**Authors:** Emily Saurman, David Lyle, Sue Kirby, Russell Roberts

**Affiliations:** 1 Broken Hill University Department of Rural Health, University of Sydney, Broken Hill, NSW 2880, Australia; E-Mails: david.lyle@health.nsw.gov.au (D.L.); sue.kirby@health.nsw.gov.au (S.K.); 2 University of Sydney, Orange, NSW 2800, Australia; E-Mail: rustleroberts@hotmail.com

**Keywords:** mental health, telemedicine, emergency medical services, efficiency, program development, Australia

## Abstract

The Mental Health Emergency Care-Rural Access Program (MHEC-RAP) is a telehealth solution providing specialist emergency mental health care to rural and remote communities across western NSW, Australia. This is the first time and motion (T&M) study to examine program efficiency and capacity for a telepsychiatry program. Clinical services are an integral aspect of the program accounting for 6% of all activities and 50% of the time spent conducting program activities, but half of this time is spent completing clinical paperwork. This finding emphasizes the importance of these services to program efficiency and the need to address variability of service provision to impact capacity. Currently, there is no efficiency benchmark for emergency telepsychiatry programs. Findings suggest that MHEC-RAP could increase its activity without affecting program responsiveness. T&M studies not only determine activity and time expenditure, but have a wider application assessing program efficiency by understanding, defining, and calculating capacity. T&M studies can inform future program development of MHEC-RAP and similar telehealth programs, both in Australia and overseas.

## 1. Introduction

The application of telehealth technologies for mental health care is not new and has been used to provide various clinical services and support, supervision and learning, and administrative activities from a distance for decades both in Australia and abroad [1,2,3,4,5,6,7,8,9]. It is promoted in rural and remote communities to improve access to specialists and the timely and effective provision of quality care [10,11,12,13]. Most specialist care in rural Australia, from cardiologists to psychiatrists, is provided by visiting clinicians. Only 16% of all health specialists consult in rural or remote communities where 30% of the Australian population resides [14,15,16].

The provision of specialist mental health care in rural and remote communities is hindered by vast distances, geographic isolation, and ongoing workforce shortages [14,15,17,18,19]. The management of mental health emergencies in communities without ready access to specialist expertise can result in a delay of referral and/or diagnosis due to underestimating the condition, or the unnecessary transfer of some patients out of their community [18,20,21]. The timely intervention with specialist mental health care can reduce patient distress and benefit patient outcomes [18,20,22,23].

**Figure 1:**
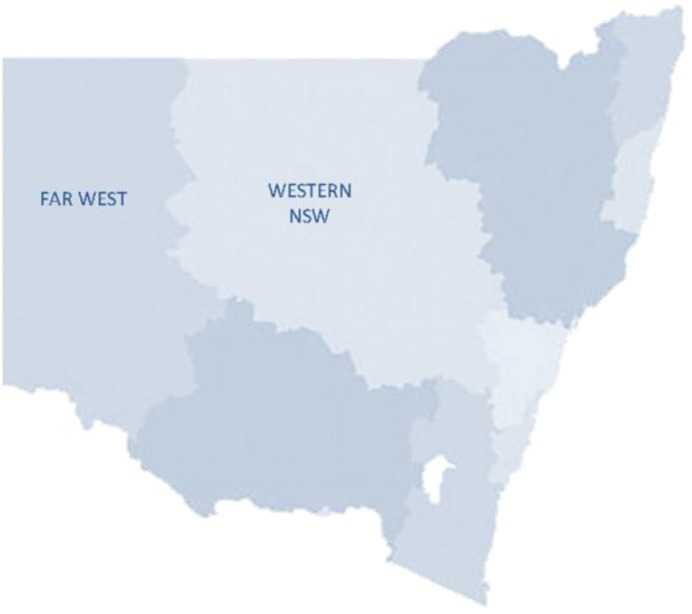
Map of New South Wales (NSW) Local Health Districts (LHD)—Far West LHD and Western NSW LHD identified.

The Mental Health Emergency Care-Rural Access Program (MHEC-RAP); a 24/7 rural emergency telepsychiatry program, is contacted through the state freecall mental health telephone access line (1800011511) and aims to improve access to specialist emergency mental health care [24,25,26]. It has been operating since February 2008 and provides a dedicated team of mental health specialists who offer timely information and support, emergency telephone triage assessments, and video assessments for anyone needing urgent mental health care across the Far West and Western NSW Local Health Districts; a population of approximately 300,000 living across 445,000 kms^2^ or 55% of the state (Figure 1).

Evaluation findings have already been reported on service activity and program reach [27,28]. The decision to fund the program represents an opportunity cost that needs to be considered both in terms of improved access to specialist services and program efficiency. Emergencies are difficult to predict, so an emergency care model needs to balance responsiveness and efficiency to provide value for money. This article presents the findings of a time and motion (T&M) study that aimed to determine the amount of time required for MHEC-RAP activities and to assess program efficiency based on the model design and current clinical practice.

## 2. Method

A T&M study was conducted to assess the time taken to complete MHEC-RAP activities and to use these data to inform program development. This was part of a larger study that aimed to describe the structure of MHEC-RAP, how it functions, its processes, and efficiency [29]. T&M studies are usually associated with work improvement strategies. They are often used for continuous and independent observations of clinical work and have been found to be a more reliable method than work sampling, self-reporting, or questionnaires for documenting process [30,31,32]. Detailed findings of the observational component of this study, reporting the model design will be published separately.

The “STAMP” (Suggested Time And Motion Procedures) method was used to report our findings. STAMP is a checklist developed for T&M studies to improve consistency in research and reporting covering areas from the intervention, design, and analysis to the observer and subject [30]. For this study we did not consider cost effectiveness nor the relative efficiency of program delivery compared with other program approaches.

The study took place over 15 consecutive days in April 2013 at the MHEC-RAP facility on the Bloomfield Campus in Orange, NSW. The observations were conducted by ES as part of her PhD Candidature. She was also part of an initial study of MHEC-RAP in 2008 during which time she was co-located with the MHEC-RAP team; this provided useful background knowledge of the program [33]. ES only took a break during an observation period to use the restroom or communicate with her PhD supervisors; food and drink were consumed with the team in the “control room”—the main room from which the team operates.

At the time of the study, MHEC-RAP operated using a 12-h shift schedule with two clinicians rostered on each shift (day/AM and night/PM). A third clinician worked on an overlapping shift each day between 1300–2100. All 17 members of the MHEC-RAP team were invited to participate in the study, including casual staff. As MHEC-RAP is a 24/7 program, observations were scheduled to accommodate non-consenting staff and to ensure observation of day and night shifts across all days of the week. ES observed seven 12-h shifts (3 day/4 night) and four morning sessions (0700–300) on 11 of 15 (73%) possible days during the study period.

Data were collected in the form of detailed notes (who was acting and what they were doing) and timed activity records (start and finish times of each activity, hour:minute). Activity data were also extracted from the MHEC-RAP call logs and the clinical service dataset for the study period. All notes, activity data, and times were entered on a form created for the purpose, and transcribed into EXCEL spreadsheets for future analysis. Timed activity records were from the initiation of the activity and included all aspects of the activity (including administrative tasks, such as paperwork) through to the completion of the case. For activities that involved more than one rostered clinician, a summary measure of person-time was calculated.

For these analyses, MHEC-RAP activities were grouped, based on findings from the observational study, into:

- clinical services (the telephone triage and video assessments),
- information services (general information, support, and advice), and
- other program activity (handover, case review, and other case related activity not clinical or information related).

Geometric means and medians were calculated for all analyses because MHEC-RAP activity data were skewed. The Geometric mean normalizes the range of numbers. The median is the middle value of the range and is a robust statistic to report with skewed data.

Three clinical service activities (two telephone triage only and one with video assessment) were managed across shifts so we used mean substitution for the unobserved tasks to calculate the total activity time. Actual time spent on activities during a shift was compared with an estimate based on the geometric mean times to identify outlier observations for more detailed analysis and to inform the use of T&M findings to estimate program efficiency.

The total available time for MHEC-RAP service provision was calculated as follows: for each 12-h shift two hours were subtracted for meal and work breaks for each clinician, and one hour for the 8-h shift. This resulted in 2820 min of available time for MHEC-RAP service provision each 24-h.

Program efficiency was estimated for the study period (to compare AM and PM shifts) and for a 12 month period using 2011 activity data which was previously collected for the evaluation [27,28]. These data were summarized graphically and used medians because data were skewed. Program efficiency was calculated using the geometric mean times and the activity data; the formulas used, and examples, are in Table 1. All data were analysed using EXCEL and Wessa [34,35].

**Table 1:** The formulas used to calculate program efficiency of the Mental Health Emergency Care-Rural Access Program.

Variable	Description	Formula and Example Calculation
n	number of days in time period	n
		for example: 31 days ^
m	minutes available in time period	(n × 2820)
		for example: months of 31 days × 2820 = 87,420
CLINICAL SERVICES		
cs	number of clinical services for time period	cs
		for example: 255
va	number of video assessments for time period	va
		for example: 71
TVA	total time conducting video assessment	(va × 126.35)
		for example:71 × 126.35 = 8970.85
tr	number of telephone triages for time period	(cs-va)
		for example: 184
TTR	total time conducing telephone triage	(tr × 64.81)
		for example:184 × 64.81 = 11,925.04
TCS	total time conducting clinical services	(TVA + TTR)
		for example: 8970.85 + 11,925.04 = 20,895.89
W	proportion of total available time conducting clinical services for time period	(TCS/m) or ((TVR = TTR)/m)
		for example: month of 31 days = 23.90
INFORMATION SERVICES		
in	number of incoming case related calls (not related to cs) for time period	in
		for example: 782
TIN	total time conducting incoming calls	(in × 2.51)
		for example:782 × 2.51 = 1962.82
ot	number of outgoing case related calls (not related to cs) for time period	ot
		for example: 193
TOT	total time conducting outgoing calls	(ot × 1.68)
		for example:193 × 1.68 = 324.24
TIS	total time conducting information services	(TIN + TOT)
		for example:1962.82 + 324.24 = 2287.06
x	proportion of total available time conducting information services for time period	(TIS/m) or ((TIN + TOT)/m)
		for example: month of 31 days = 2.62
OTHER PROGRAM ACTIVITIES		
oa	time conducting other program activities (handover, casereview, otheractivities)	handoverAM = minsx2staff; handoverPM = minsx2staff; casereviewAM = minsx2staff; otheractivitiesAM = mins; otheractivitesPM = mins
oawd	weekday variation	handover(AM + PM) + casereviewAM + otheractivities(AM + PM) for example: 25.42 + 13.00 + 81.04 + 14.50 + 3.33 = 137.29
oawe	weekend variation	handover(AM + PM) + otheractivities(AM + PM) for example: 25.42 + 13.00 + 14.50 + 3.33 = 56.25
TOA	total time conducting other activities	(oawd xn) + (oawe xn) for example: month of 31 days = (137.29 × 21) + (56.25 × 10) = 3445.59
y	proportion of total available time conducting other program activity for time period	(TOA/m) or ((oawd xn) + (oawe xn)/m) for example: month of 31 days = 3.94
TOTAL TIME		
TT	total time spent in activity	(TCS + TIS + TOA) for example: 20,895.89 + 2287.06 + 3445.59 = 26,628.54
z	proportion of total available time spent in activity for time period	(TT/m) or (w + x + y) for example: month of 31 days = 30.46

^ Numbers in the examples are those for January 2011.

## 3. Results

### 3.1. Time and Motion

There were 299 activities observed during the study period—18 clinical services, 239 information services, and 42 other program activities. Information services accounted for 80% of overall activity in terms of numbers, but only 21% in terms of time with each telephone call lasting 2–3 min on average. The clinical services accounted for 6% of all activities and 50% of the time for those activities.

It took 65 min on average to complete a telephone triage, increasing twofold to 126 min when a video assessment was organized as well. Time required for the clinical care component ranged from a minimum of 8 min for telephone triage only to a maximum of 96 min with a video assessment, averaging 17 and 47 min respectively. Arranging care required 4 to 5 min on average, however in one instance this took 51 min. Completing the associated clinical paperwork accounted for more than half the time taken to deliver a clinical service—63% for telephone triage only and 52% with a video assessment (Table 2).

**Table 2:** Time expenditure for the Mental Health Emergency Care-Rural Access Program by activity, 15 consecutive days in April 2013.

Activity	Number Observed	Geometric Mean Time			
		Total Time Mins (Range)	Clinical Care Mins (Range)	Arranging Care * Mins (Range)	Paperwork ** Mins (Range)
CLINICAL SERVICES					
Telephone Triage only	8	64.81 (26–165)	17.35 (8–75)	4.02 (2–9)	39.13 (13–152)
with Video Assessment	10	126.35 (49–309)	47.10 (20–96)	5.01 (2–51)	58.79 (12–174)
INFORMATION SERVICES					
Incoming Call	132	2.51 (1–36)	NA ^^	NA	NA
Outgoing Call	107	1.68 (1–15)	NA	NA	NA
OTHER PROGRAM ACTIVITY					
Handover(AM) ***	7	25.42 (12–54)	NA	NA	NA
Handover(PM) ***	4	13.00 (4–24)	NA	NA	NA
Case Review(AM) ***	6	81.04 (40–168)	NA	NA	NA
Other Activity(AM) ^	16	14.50 (1–45)	NA	NA	NA
Other Activity(PM) ^	9	3.33 (1–8)	NA	NA	NA

* Arranging Care is time spent contacting referral and transport services; ** Paperwork is the time spent completing the required documentation (Triage, Assessment, Discharge Forms); *** Reporting person minutes based on 2 persons participating per shift; ^ Other activity includes discussions, following-up on a case, completing data logs, and other case related activities that are not clinical or information service related; ^^ NA = Not Applicable.

Case reviews were conducted each weekday (AM shift) and involved the supervising psychiatrist, the two rostered MHEC-RAP clinicians, and the Nurse Unit Manager. This activity required 81 person-min on average. Clinical handover occurred twice a day between shifts, and took 25 person-min on average for the AM shift and 13 person-min for the PM shift.

**Figure 2:**
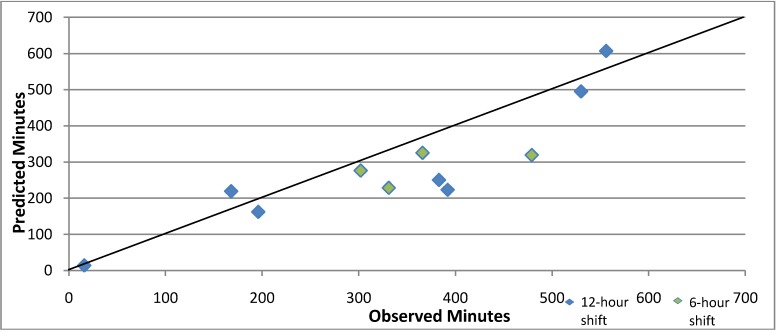
Scatterplot comparing the observed and predicted times (in minutes) conducting Mental Health Emergency Care-Rural Access Program Activities during 12-h shifts (blue) and 6-h shifts (green), April 2013.

We compared the time spent on activities during the observation period with an estimate based on the mean times (Figure 2). The predictive model underestimated the actual times, (mean difference—54.55 min t_10df_ = 2.35, *p* < 0.05). We reviewed the four shifts whose actual times exceeded the predicted values by more than 50%. For three, between 57% and 100% of the difference was accounted for by the additional time spent completing paperwork in excess of the average, and for the fourth, which had only one clinical service, all of the difference was attributed to a lengthy case review and other program activities.

### 3.2. Program Efficiency

**Figure 3:**
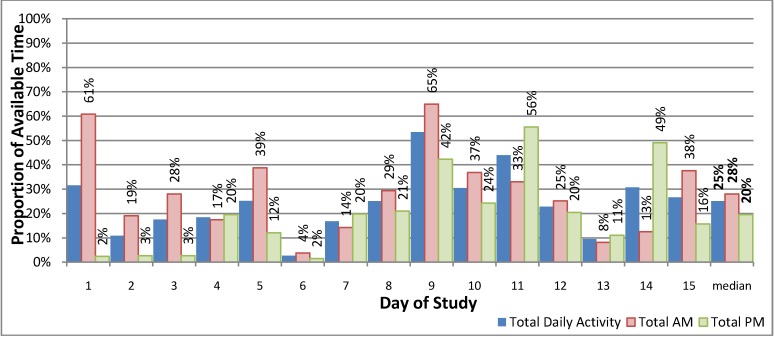
The proportion of available time in active service provision—Mental Health Emergency Care-Rural Access Program, 15 consecutive days in April 2013.

The estimated time spent providing MHEC-RAP activities varied between the AM and PM shifts during the study period (Figure 3). This represented a median of 28% of the available time for the AM shift (range: 4%–65%) and 20% of the PM shift (range: 2%–56%). However when case review was excluded from the weekday AM shifts, the median was 22% (range: 4%–59%) indicating similar amounts of time was spent on clinical service activity for both the AM and PM shifts.

**Figure 4:**
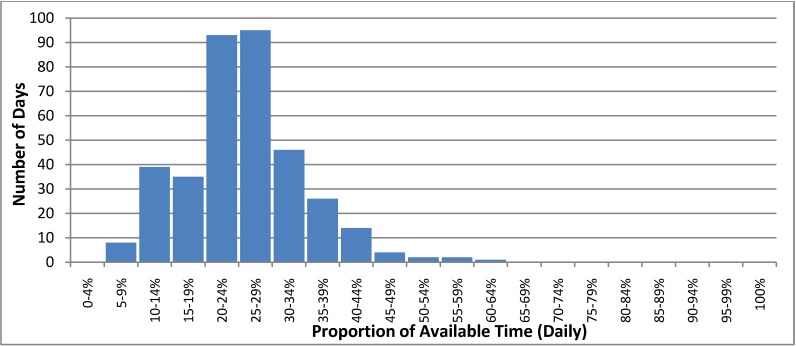
The proportion of available time spent providing Mental Health Emergency Care-Rural Access Program activities on a daily basis in 2011.

In 2011, we estimated that a median of 26% (range 7%–61%) of the available time was spent providing MHEC-RAP activities on a daily basis. The estimated time spent on MHEC-RAP activities exceeded 50% of the available time for only 5 days in 2011. For 175 days that year, less than a quarter of the available time each day was spent providing program activities (Figure 4).

## 4. Discussion

This T&M study provided valuable information, not otherwise available, about the amount of time required for MHEC-RAP activities and program efficiency. It emphasized the importance of telephone triage and video assessment activity in relation to program efficiency (mean time required of 65 min and 126 min respectively for each activity). Even at low levels of activity they accounted for the majority of time spent on service delivery and thus represent the most important determinant of program efficiency. One area that would benefit from more efficient service delivery is the completion of paperwork, which accounted for at least 50% of the time required for a clinical service. This activity showed significant variability and has the potential to impact program performance if it is not effectively managed.

The T&M study findings have wider application. During 2011, the estimated time required for MHEC-RAP activities accounted for about one-quarter of the available time and only exceeded half of the available time on five days that year. This indicated that the program had capacity to support a higher level of clinical service activity. Currently, there is no efficiency benchmark for emergency telehealth mental health care programs. T&M studies could be used to derive benchmarks for efficient service delivery and inform the development of similar telehealth programs, both in Australia and overseas. Like other emergency services, MHEC-RAP must manage efficiency against capacity and based on our data, an initial increase in activity to achieve a median of 40% of the available time in active service provision should be possible without adversely affecting program responsiveness. Increased activity would result in greater program efficiency and further address the aim to improve access to the service. For MHEC-RAP, this could be achieved by promoting greater use of the program within the existing service footprint or extending the program reach to additional communities in adjacent regions.

Future research could provide more detailed breakdown of the different elements of MHEC-RAP activities including the time taken to coordinate the admission and transportation of patients requiring specialist care in a Mental Health Inpatient Unit, for supervision and support of generalist staff to manage patients in the local ED, and support of community mental health staff in smaller centres. Additional investigations could review MHEC-RAP against the standards for NSW Health Mental Health Telephone Triage Services such as the time to answer a call and compare the time taken to complete telephone triage and video assessment by referral source to provide a greater understanding of program performance [36].

The design specifications for similar telehealth programs, including geographical reach and minimum staffing levels, influence demand for the service and their capacity to manage workload. Nonetheless, getting program efficiency right is primarily a function of maximizing the number of emergencies referred to the service, on average, over an extended period, with adjustments for the variability in numbers on a daily basis to maintain service responsiveness. Further work is required to generate metrics that would enable benchmarks to be established and calculations to be made that set realistic program efficiency targets and assist service planning.

## 5. Limitations and Strengths

The study is based on a small number of clinical services collected during a period of low program activity which reduced the precision of study estimates. The lack of time pressure on some shifts may have influenced the amount of time taken to complete certain activities such as the completion of paperwork, resulting in prolonged times. Program efficiency measures were derived from routinely collected data accessed through MHEC-RAP service records for 2011. Data were limited to a daily count of clinical activities and a summary of in-coming and outgoing calls on a monthly basis restricting our analysis to daily measures of program efficiency and potentially reducing the variance of those daily estimates.

The use of well accepted and robust T&M methods, based on independent observation of activity and administered by one observer, are study strengths that support the validity of the findings.

## 6. Conclusions

MHEC-RAP is not the first telehealth service to be developed in Australia or overseas, but it is the first model to combine 24-h availability and access to regionally-based emergency specialist care using telehealth technology. The T&M study provided valuable information, not otherwise available, about the amount of time required for MHEC-RAP processes and program efficiency. The study emphasized the importance of telephone triage and video assessment activity in relation to program efficiency and that the program had capacity to support a higher level of clinical service activity. The T&M study findings have wider application and could be used to derive benchmarks for efficient service delivery and inform the development of similar telehealth programs, both in Australia and overseas.
